# Deep learning magnetic resonance imaging predicts platinum sensitivity in patients with epithelial ovarian cancer

**DOI:** 10.3389/fonc.2022.895177

**Published:** 2022-11-23

**Authors:** Ruilin Lei, Yunfang Yu, Qingjian Li, Qinyue Yao, Jin Wang, Ming Gao, Zhuo Wu, Wei Ren, Yujie Tan, Bingzhong Zhang, Liliang Chen, Zhongqiu Lin, Herui Yao

**Affiliations:** ^1^ Guangdong Provincial Key Laboratory of Malignant Tumor Epigenetics and Gene Regulation, Medical Research Center, Sun Yat-sen Memorial Hospital, Sun Yat-sen University, Guangzhou, China; ^2^ Department of Gynecological Oncology, Sun Yat-sen Memorial Hospital, Sun Yat-sen University, Guangzhou, China; ^3^ Department of Medical Oncology, Sun Yat-sen Memorial Hospital, Sun Yat-sen University, Guangzhou, China; ^4^ Faculty of Medicine, Macau University of Science and Technology, Macao, Macao SAR, China; ^5^ Phase I Clinical Trial Centre, Sun Yat-sen Memorial Hospital, Sun Yat-sen University, Guangzhou, China; ^6^ Cells Vision Medical Technology Inc., Guangzhou, China; ^7^ Department of Radiology, Sun Yat-sen Memorial Hospital, Sun Yat-sen University, Guangzhou, China; ^8^ Breast Tumor Centre, Sun Yat-sen Memorial Hospital, Sun Yat-sen University, Guangzhou, China

**Keywords:** deep learning, magnetic resonance imaging, platinum sensitivity, epithelial ovarian cancer, non-manually segmented

## Abstract

**Objective:**

The aim of the study is to develop and validate a deep learning model to predict the platinum sensitivity of patients with epithelial ovarian cancer (EOC) based on contrast-enhanced magnetic resonance imaging (MRI).

**Methods:**

In this retrospective study, 93 patients with EOC who received platinum-based chemotherapy (≥4 cycles) and debulking surgery at the Sun Yat-sen Memorial Hospital from January 2011 to January 2020 were enrolled and randomly assigned to the training and validation cohorts (2:1). Two different models were built based on either the primary tumor or whole volume of the abdomen as the volume of interest (VOI) within the same cohorts, and then a pre-trained convolutional neural network Med3D (Resnet 10 version) was transferred to automatically extract 1,024 features from two MRI sequences (CE-T1WI and T2WI) of each patient to predict platinum sensitivity. The performance of the two models was compared.

**Results:**

A total of 93 women (mean age, 50.5 years ± 10.5 [standard deviation]) were evaluated (62 in the training cohort and 31 in the validation cohort). The AUCs of the whole abdomen model were 0.97 and 0.98 for the training and validation cohorts, respectively, which was better than the primary tumor model (AUCs of 0.88 and 0.81 in the training and validation cohorts, respectively). In k-fold cross-validation and stratified analysis, the whole abdomen model maintained a stable performance, and the decision function value generated by the model was a prognostic indicator that successfully discriminates high- and low-risk recurrence patients.

**Conclusion:**

The non-manually segmented whole-abdomen deep learning model based on MRI exhibited satisfactory predictive performance for platinum sensitivity and may assist gynecologists in making optimal treatment decisions.

## Introduction

The standard treatment of epithelial ovarian cancer (EOC) comprises debulking surgery and platinum-based chemotherapy (1). Although a high remission rate can be achieved, 20%–30% of patients received multiple cycles of toxic therapy before platinum resistance was identified and delayed the initiation of therapy with effective agents, which turned out to be a major impediment to improved outcomes (2). At the same time, platinum sensitivity is a simple index to screen out populations sensitive to poly (ADP-ribose) polymerase inhibitors (PARPi) (3, 4), and this prediction may reduce the unnecessary enrollment of patients into various clinical trials. Accordingly, if platinum sensitivity could be reliably predicted, patients would gain more benefits from precision therapy (5). However, classic clinical indicators such as CA125 and tumor immunohistochemistry possess limited predictive power (6). Nowadays, biopsies followed by mutation profiling or surgical resections have become a standard and informative procedure (7). However, high cost, invasiveness of the approaches, intra-tumoral heterogeneity, and repeated tumor sampling enormously limit the applicability of such molecular testing, raising much concern regarding the cost–benefit balance (8).

Contrastingly, MRI is a non-invasive method to detect tumors by obtaining the size, location, and other basic imaging information. A study that recruited 125 participants with EOC found that the changes in apparent diffusion coefficient (ADC) after chemotherapy are indicative of the response to platinum (9). Additionally, radiomics based on MRI have been developed to recognize different subtypes of EOC (i.e., benign, borderline, malignant) (10) and 3-year recurrence prediction was also effective (11). However, conventional radiomic methods require manual detection, segmentation, and extraction, which is time-consuming, tedious, and susceptible to manual operation errors and may be unsuitable for general practice at this time (12, 13). This highlights the urgent need for the development of new radiomic methods to predict the platinum sensitivity of patients with EOC.

Deep learning with powerful algorithms greatly reduce unnecessary labor and display effectiveness in applications of medical imaging (12, 14), for instance the screening of breast cancer (15), diagnosis of cataracts (16) and Alzheimer’s disease (17), ALK-TKI or radiation therapy in patients with lung cancer (18, 19). In this study, we aimed to develop a deep learning model to predict platinum sensitivity of patients with EOC based on MRI before the initial intervention.

## Materials and methods

### Patients and study design

This study followed the STARD reporting guidelines and the Helsinki Declaration. The institutional review boards of the Sun Yat-sen Memorial Hospital approved this retrospective study with identified data [Institutional Review Board (IRB) no. SYSEC-KY-KS-2020-072] and issued a waiver for written consent. This study was registered with ClinicalTrials.gov, number NCT04511481. Data generated or analyzed during the study are available from the corresponding author on request.

**Figure 1:**
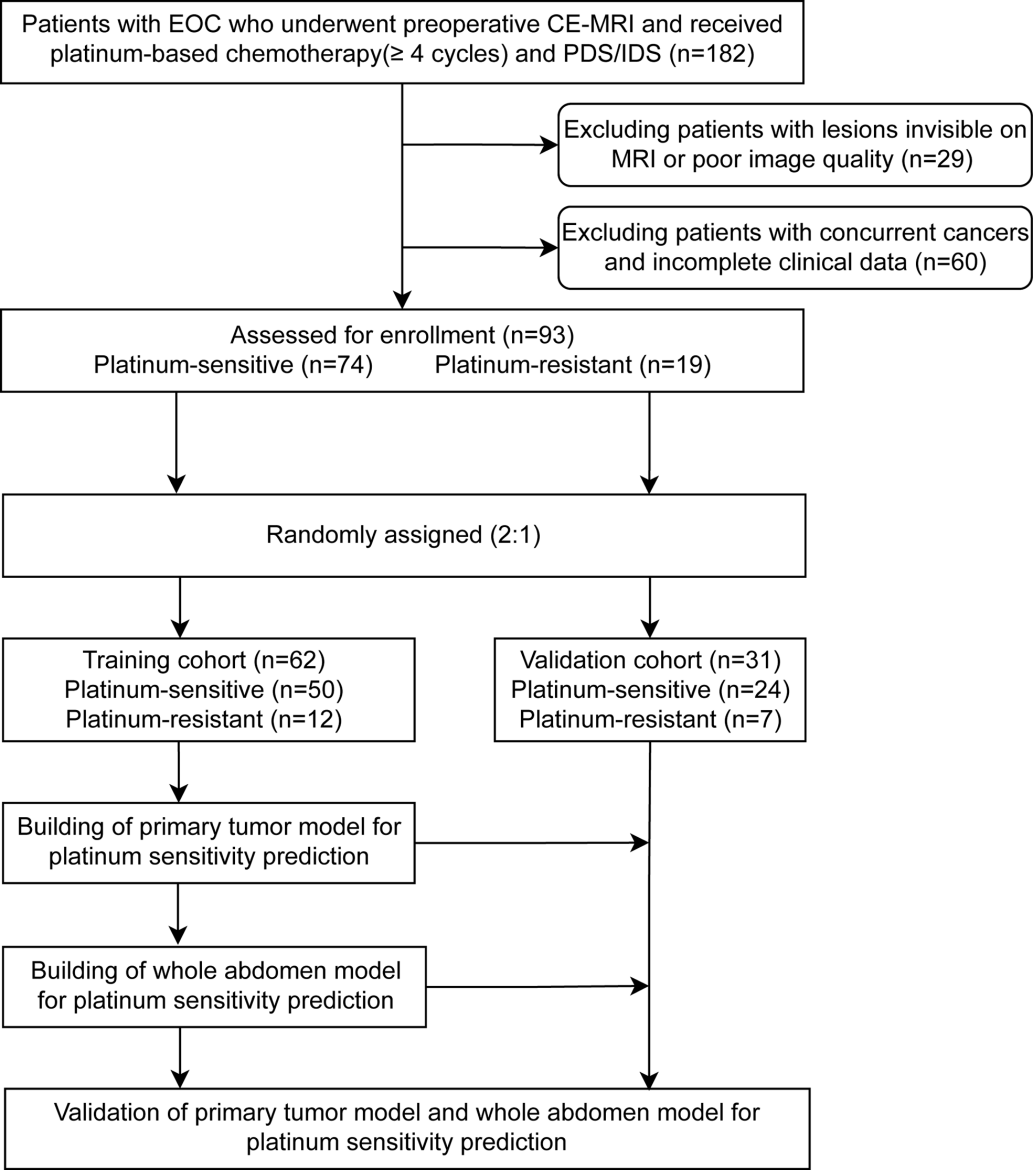
Patients’ recruitment and study design. A total of 93 patients with preoperative magnetic resonance imaging from the Sun Yat-sen Memorial Hospital were enrolled. The two representative deep learning models (whole abdomen model and primary tumor model) were constructed and validated with the same training and validation cohorts, then compared for performance. EOC, epithelial ovarian cancer; CE-MRI, contrast-enhanced magnetic resonance imaging; IDS, interval debulking surgery; PDS, primary debulking surgery.

Ninety-three patients from the Sun Yat-sen Memorial Hospital from January 2011 to January 2020 were enrolled and randomly assigned (2:1) to the training (n = 62) and validation cohorts (n = 31) (**Figure 1**). Inclusion criteria: (i) pathologically confirmed EOC; (ii) whole abdomen contrast-enhanced MRI within 2 weeks before the initial intervention (MRI detailed in **Supplementary Methods S1** and **Table S1**); (iii) received cisplatin/carboplatin chemotherapy (≥4 cycles) in the initial treatment or switched chemotherapy scheme due to primary platinum-refractory; and (iv) received either primary debulking surgery (PDS) or interval debulking surgery (IDS) (Residual disease <1 cm). Exclusion criteria: (i) cancer lesions invisible on MRI; (ii) poor quality MRI (e.g., with motion or artifacts) as assessed by two radiologists (ZW and ZC); (iii) concurrent cancers; and (iv) unavailability of complete clinical data.

Tumor response to chemotherapy was assessed by the RECIST 1.1 criteria before PDS; patients were followed up according to the NCCN guideline. The date of the last follow-up was 1 June 2021.

### Study endpoints

The primary endpoint was the platinum-free interval (PFI). According to the PFI, patients with ovarian cancer were divided into platinum-resistant (PFI <6 months) and platinum-sensitive (PFI ≥6 months). The secondary endpoint was progression-free survival (PFS), calculated from the first date of the initial intervention to disease progression or to the last progression-free date for those censored. Recurrences and distant metastases were confirmed by clinical, biochemical, or radiographic evidence or by biopsy findings.

### Image pre-processing

**Figure 2:**
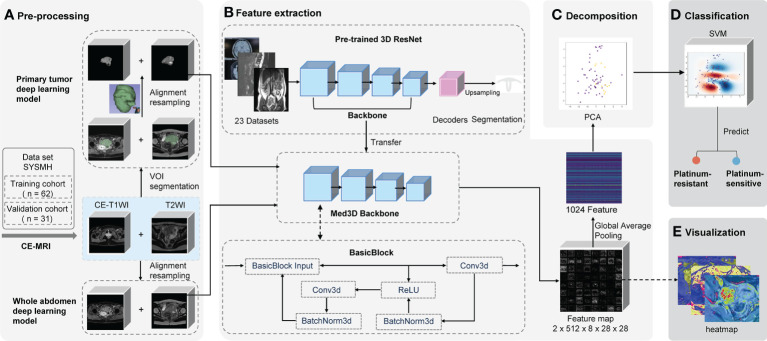
Schematic of two deep learning frameworks. The primary tumor model uses the primary tumor in CE-T1WI and T2WI sequences as the VOI with manual segmentation by radiologists. Contrastingly, the whole abdomen model uses the entire abdomen volume as VOI without any segmentation or delineation by hand. VOI on CE-T1WI and T2WI sequences were prepared for **(A)** pre-processing, which consisted of image segmentation (or not), registration, and normalization; **(B)** the backbone of the pre-trained 3D ResNet network was transferred to extract features and a global average pooling layer was added so that 1,024 features could be extracted from each patient; **(C)** the PCA was used for decomposition; **(D)** the platinum sensitivity prediction model was constructed using SVM; **(E)** the feature map that the last convolutional layer output was generated as heat maps for visualization. CE-T1WI, contrast-enhanced T1-weighted imaging; T2WI, T2-weighted imaging; VOI, volume of interest; MRI, magnetic resonance imaging; PCA, principal component analysis; SVM, support vector machine.

We built prediction models based on different algorithms, MRI sequences, and volumes of interest (VOIs). The two representative deep learning models (whole abdomen model and primary tumor model) were constructed and validated with the same training and validation cohorts. The difference between the two models was the VOI in the preprocessing part (**Figure 2A**).

The whole abdomen model took the entire abdomen volume on axial CE-T1WI sequence (venous phase) and axial T2WI (acquired before the injection of contrast medium) as the VOI to be the input of the model without any segmentation and delineation by hand, which is the most significant difference to the primary tumor model. The, the sequences of an individual patient were first aligned into the same coordinate system in reference to the CE-T1WI sequence. Then, pixels outside of the human body area, which was defined by OTSU thresholding, were filled with Gaussian noise as in the original implementation of the Med3D network (20, 21).

The primary tumor model took the primary tumor area as the VOI on axial CE-T1WI and T2WI sequences. First, sequences were loaded into 3D Slicer software (version 4.10.2) in NRRD format and were then manually segmented and delineated slice-by-slice by two radiologists (ZW and ZC) independently as precisely as possible. The radiologists were blinded to prognostic information. The data was then reviewed by two radiologists (MG and ZW) from the same hospital, each of whom had more than 15 years of experience in gynecologic oncology radiology and was mainly responsible for the VOI assessment. Any disagreements were resolved by consensus among the four radiologists.

To eliminate the interference, all values from the areas outside of the VOIs were removed and filled with Gaussian noise, similar to the whole abdomen model (**Figure 2A**). Finally, spatial and intensity normalization was applied to these VOIs (see **Supplementary Methods S2**).

### Model building and validation

Features were extracted from the input data using a feed-forward neural network. It was pre-trained on a few medical imaging datasets to learn to capture useful information from medical images. Through the power of transfer learning, we are able to transfer the knowledge that the model has acquired from one task and apply it to the other. This means that the model does not need to learn everything from scratch every time. The neural network uses 3D convolution operations as its core structure. Hierarchically, deeper layers will produce more complicated features. For example while shallow layers may capture edges or brightness information, deeper layers may capture texture or even abnormality information. The model uses a residual structure, so that information extracted by the shallow layers will not be “forgotten” during the feeding forward through so many layers.

A pre-trained convolutional neural network Med3D (Resnet 10 version) was transferred as the feature encoder to extract features (**Figure 2B** and **Supplementary Methods S3**) from the chosen VOI. Following the feature extraction on corresponding VOIs, we applied principal component analysis (PCA) to perform the decomposition (**Figure 2C** and **Supplementary Methods S4**). It denoised and compressed them into smaller dimensions. Finally, a support vector machine (SVM) was fitted to the data produced by the PCA to achieve the classification of how likely a patient is to be platinum-sensitive (**Figure 2D** and **Supplementary Methods S5**).

The validation cohort’s data was left completely untouched throughout the training process. In the building of the model pipeline, cross-validation was used to evaluate the model performance and to decide on the hyperparameters. All programs were run on Python version 3.6.8.

### Feature visualization

Feature maps were acquired from the output of the last convolution layer right before global average pooling. All the feature maps were averaged to get a heat map to determine which areas of the image were the most active and deserved the model’s focus (**Figure 2E**).

### Statistical analysis

When assessing clinical characteristics, categorical variables were compared using the χ^2^ test, and continuous variables were compared using the independent *t*-test. Receiver operating characteristic curve (ROC) analysis was used to determine the optimal threshold for maximizing prediction accuracy and to assess and evaluate the performance of models. Five-fold cross-validation was employed to compare robustness. The PFS was calculated using the Kaplan–Meier method, and the log-rank test, hazard ratios (HRs), and 95% confidence intervals (Cls) were calculated using a Cox regression analysis. The optimal cutoff values were generated with the R package ggsurvimier. Moreover, the calibration curve and decision curve analysis (DCA) were plotted to assess the whole abdomen model. P-values of less than 0.05 were considered significant, and all tests were 2-tailed. Statistical analyses were conducted with Python 3.6.8, and R version 3.6.1.

## Results

### Characteristics of the patients

A total of 93 patients with EOC from the Sun Yat-sen Memorial Hospital were eligible for this study (**Figure 1**). **Table 1** shows the clinicopathological characteristics of patients from the training cohort (n = 62) and the validation cohort (n = 31), 50 (81%) of the 62 patients in the training cohort and 24 (77%) of the 31 patients in the validation cohort were platinum-sensitive. Hyperthermic intraperitoneal chemotherapy (HIPEC) was administered to 24 (39%) of the patients in the training cohort and 12 (39%) of the patients in the validation cohort. The median follow-up was 21.0 months (interquartile range [IQR] 13.7–33.2) for patients in the training cohort and 23.2 months (IQR 15.2–43.3) for the validation cohort. The clinicopathological variables between the training cohort and the validation cohort had no statistically significant difference (*P* >.05). In the univariate logistic regression analysis, stages were found to be associated with platinum sensitivity in the training cohort (*P* <.001) (**Table S2**).

**Table 1:** Characteristics of patients in the training and validation cohorts.

Characteristic	Training cohort *n* = 62	Validation cohort *n* = 31	*p*-value^a^
Patients, No. (%)			
Age, mean (SD), year	49.4 (11.2)	52.9 (8.6)	.21
Platinum sensitivity, No. (%)			.93
Resistant	12 (20)	7 (23)	
Sensitive	50 (80)	24 (77)	
HIPEC, No. (%)			>.99
≥1 cycle	24 (39)	12 (39)	
Non	38 (61)	19 (61)	
FIGO stage^b^, No. (%)			.73
I	13 (21)	8 (26)	
II	4 (6)	3 (10)	
III	37 (60)	18 (58)	
IV	8 (13)	2 (6)	
Histologic classification, No. (%)			.32
Serous carcinoma	39 (63)	20 (65)	
Endometrioid carcinoma	9 (15)	1 (3)	
Clear cell carcinoma	5 (8)	6 (19)	
Mucinous carcinoma	2 (3)	1 (3)	
Special types	7 (11)	3 (10)	
Type of surgery, No. (%)			>.99
PDS	54 (87)	27 (87)	
IDS	8 (13)	4 (13)	
Follow-up time, months (median [IQR])	21.0 [13.7, 33.2]	23.2 [15.2, 43.3]	.44

FIGO, International Federation of Gynecology and Obstetrics; SD, standard deviation; HIPEC, hyperthermic intraperitoneal chemotherapy; PDS, primary debulking surgery; IDS, interval debulking surgery; IQR, interquartile range.

a p-values represent the difference of each clinicopathologic variable between the training and validation cohorts.

b 2018 FIGO staging.

### Deep learning models predict platinum sensitivity

The performance of the primary tumor model compared with the whole abdomen model is summarized in **Table S3**. The primary tumor model achieved areas under the receiver operating characteristic curves (AUCs) of 0.88 (95% CI, 0.79–0.97) and 0.81 (95% CI, 0.65–0.97), with accuracy (ACC) of 0.87 and 0.81, the sensitivity of 90% and 88%, and specificity of 75% and 57% in the training and validation cohorts, respectively. Five-fold cross-validation showed the specificity and sensitivity had an obviously reduced result (**Table S3** and **Supplementary Figure S1**).

**Figure 3:**
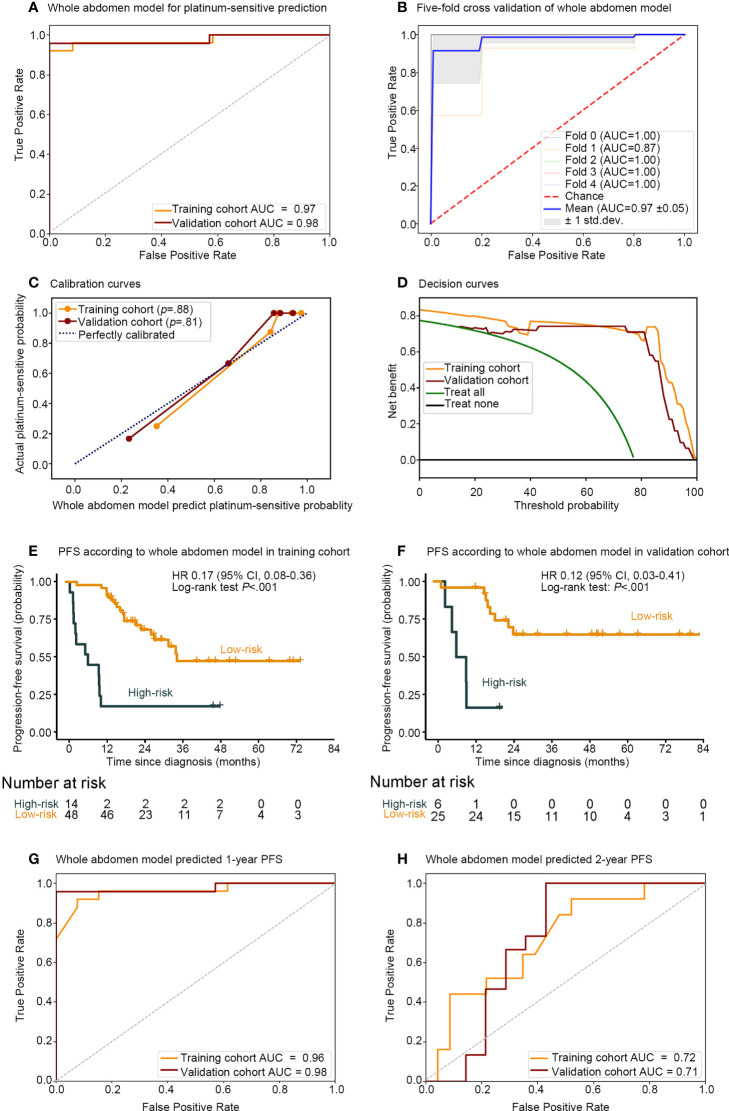
Performance of whole abdomen model including association between the whole abdomen model decision function value and the PFS. Receiver operating characteristic (ROC) curves **(A)** of whole abdomen model performance for predicting platinum sensitivity; **(B)** five-fold cross-validation, and evaluation with **(C)** calibration curve; **(D)** decision curve. Kaplan–Meier curves of PFS according to the decision function value in 93 patients split into high- and low-risk groups from both the **(E)** training and **(F)** validation cohorts by cutoff values of 0.296 and −1. ROC curves and **(G)** 1-, **(H)** 2-, year AUCs were used to assess the prognostic accuracy of the whole abdomen model. PFS, progression-free survival; ROC, receiver operating characteristic; AUC, area under the receiver operating characteristics curve; HR, hazard ratio; CI, confidence interval; P values were calculated using the unadjusted log-rank test, and hazard ratios were calculated by a univariate Cox regression analysis.

In comparison, the whole abdomen model achieved AUCs of 0.97 (95% CI, 0.93–1.00) and 0.98 (95% CI, 0.93–1.00), with ACC of 0.95 and 0.97, the sensitivity of 96% and 96%, and the specificity of 92% and 100% in the training and validation cohorts, respectively. The robustness of the whole abdomen model was verified by five-fold cross-validation, which showed that ACC, sensitivity, and specificity maintained satisfactory levels (**Figures 3A, B** and **Table S3**).

The calibration curve in **Figure 3C** indicated that the whole abdomen model was close to the perfect model and did not systematically make under- or over-predictions of platinum sensitivity, as shown by the fact that the Hosmer–Lemeshow test yielded a non-significant statistic for this model (*P* = .88 and *P* = .81 in the training and validation cohorts, respectively). The decision curve in **Figure 3D** indicates that the use of the whole abdomen model may improve clinical outcomes.

To further assess the whole abdomen model, stratified analysis was conducted (**Supplementary Figures S2–S5**). The whole abdomen model achieved AUCs in the subgroup of Hyperthermic intraperitoneal chemotherapy (HIPEC) (AUCs 1.00, 0.94), non-HIPEC (AUCs 0.94, 1.00), stages III–IV (AUCs 0.98, 1.00), serous carcinoma (AUCs 0.97, 1.00), other pathological types (AUCs 1.00, 0.95), IDS (AUCs 1.00, 1.00), PDS (AUCs 0.97, 0.96) in the training and validation cohorts, respectively. In summary, the whole abdomen model was able to maintain stable performance across different subgroups.

### Whole abdomen model for progression-free survival

A strong association between the whole abdomen model decision function value and progression-free survival (PFS) was further demonstrated by Kaplan–Meier analysis (**Figures 3E, F**). According to the decision function value, optimal cutoff values (0.296 and −1) were generated to split patients into high- and low-risk groups in the training and validation cohorts. High-risk patients achieved a lower decision function value and shorter PFS compared with the low-risk group in the training cohort (HR 0.17; 95% CI 0.08–0.36; *P* <.001, Log-rank test) and validation cohort (HR 0.12; 95% CI 0.03–0.41; *P* <.001, Log-rank test). To further substantiate the value of the whole abdomen model in prediction and prognosis, we used the model to predict 1- and 2-year PFS (**Figures 3G, H**). ROC curves indicated that in the 1-year PFS prediction, the AUCs were 0.96 and 0.98, and reduced to 0.72 and 0.71 in the 2-year PFS prediction in the training and validation cohorts, respectively.

**Figure 4:**
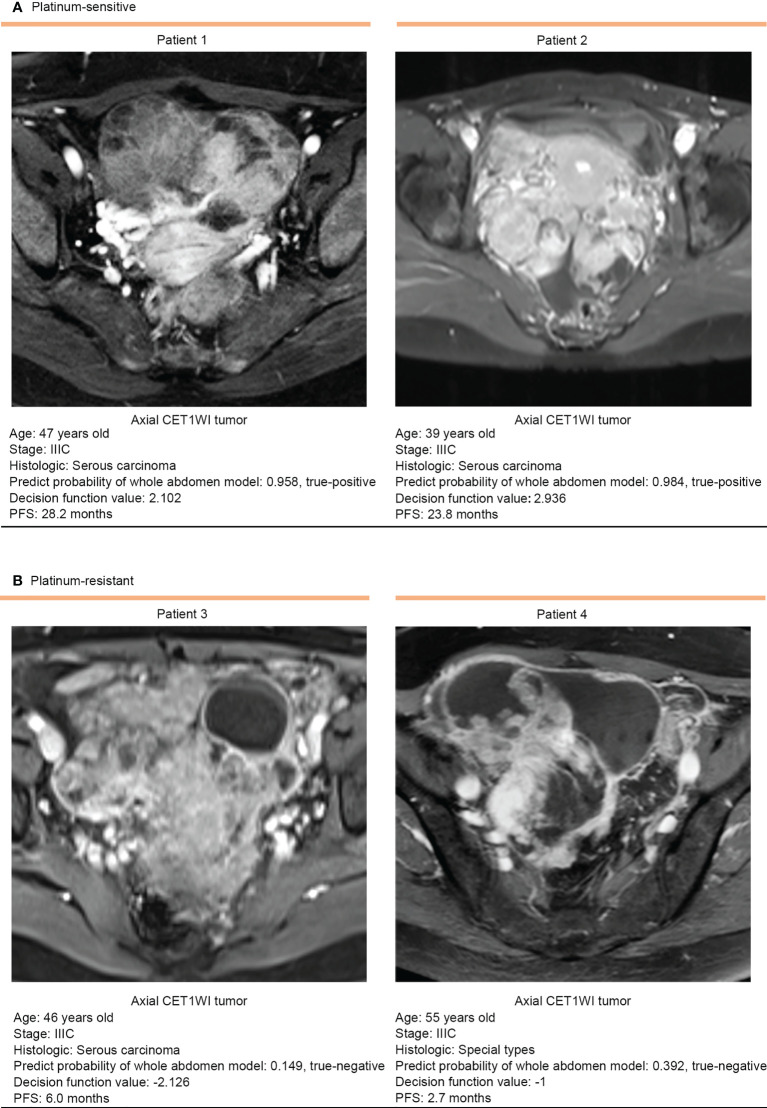
Representative prediction results of the whole abdomen model. **(A)** Patients 1 and 2 were platinum-sensitive and **(B)** patients 3 and 4 were platinum-resistant. The platinum sensitivity of patients was accurately assessed according to the decision function value by the cutoff value of 0.00. CE-T1WI, contrast-enhanced T1-weighted imaging.

As shown in **Figure 4**, patients 1 and 2 were platinum-sensitive and belonged to the low-risk group that had higher decision function values, predictive probability, and longer PFS. In contrast, patients 3 and 4 were platinum-resistant and belonged to the high-risk group that had lower decision function value, predictive probability, and shorter PFS.

**Figure 5:**
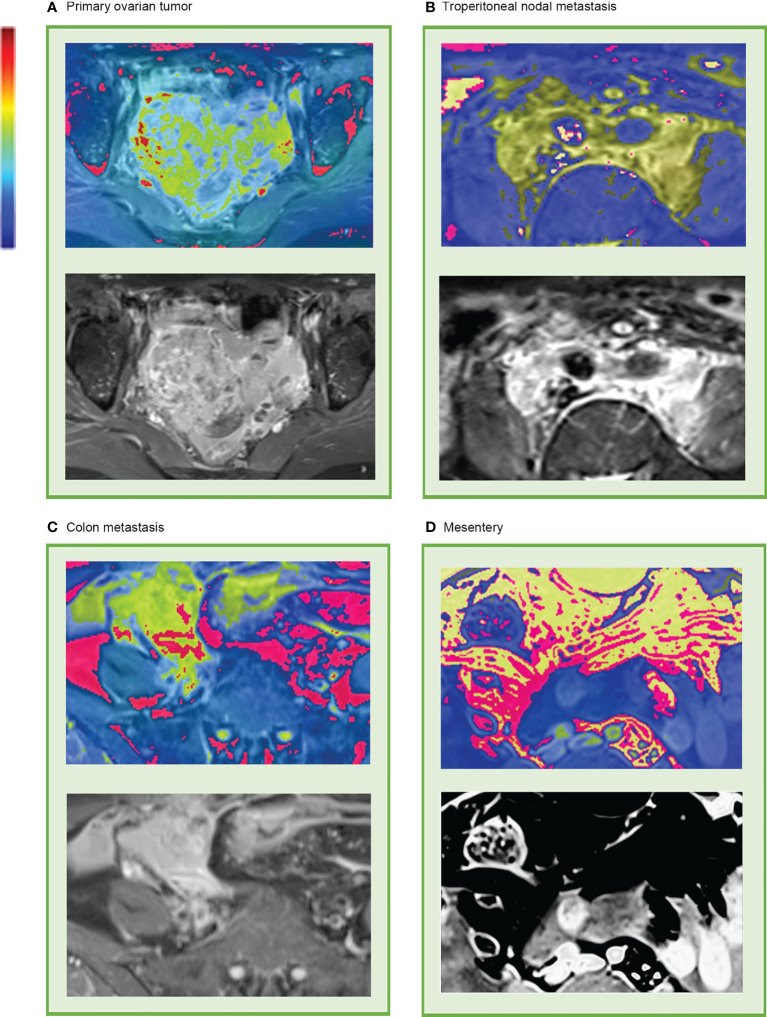
Representative heat map of deep learning feature visualization. The “rainbow” color scheme highlight showcases highly active areas. It can be observed that the highly active areas were focused on and overlapped with **(A)** primary tumor, **(B)** enlarged lymph nodes that surrounding the abdominal aorta and inferior vena cava, **(C)** colon metastasis, and **(D)** mesentery.

Although these high-level features were highly intricate, feature maps were transferred as heat maps and were observed and analyzed with the assistance of the deep learning visualization algorithms (**Figure 5**). There were some high-response areas on the heat maps, which were red, yellow, and green. The red areas appeared to be more focused on the peritoneal space; the green areas seemed to be more focused on the obvious enhancement areas that demonstrated abundant vasculature (primary tumor, enlarged para-aortic lymph nodes, colon metastasis), and the yellow areas had a typical mesenteric shape (**Figure 5D**). These patterns seemed to be observed in most heat maps.

## Discussion

This study established an end-to-end deep learning model to predict the platinum sensitivity of patients with epithelial ovarian cancer (EOC). The whole abdomen model, which took the entire volume of the abdomen on axial CE-T1WI and T2WI sequence as the volume of interest (VOI), predicted platinum sensitivity in EOC patients with high sensitivity and specificity and was validated by good calibration and decision curves. Meanwhile, the algorithm discriminated between patients with high- and low-risk recurrence and performed well in predicting 1-year PFS. In addition, the distribution patterns of high-response areas on heat maps seemed to be associated with platinum sensitivity.

As per recommendation *3.1* of the European Society for Medical Oncology consensus conference, there were no validated predictive markers of primary platinum-refractory or resistant disease (4). In contrast with previous studies, we built and validated a non-invasive whole abdomen model to provide platinum sensitivity prediction by preoperative CE-MRI, which aimed to assist in the individualized treatment of patients with EOC. Traditional radiomic analysis required time-consuming tumor delineation, which affected the reproducibility of radiomic features (19). On the contrary, since tumor boundary segmentation is not required in our whole abdomen model, deep learning features are not affected by manual operation. In this study, the input of the whole abdomen model simply required CE-T1WI and T2WI and could generate the result of the patient’s platinum sensitivity automatically within seconds without any additional workload.

ROC curves were similar in different pathological subgroups, which indicated that there may be common platinum sensitivity-related subtle imaging features in different pathologies which could be captured and discriminated by the whole abdomen model. In stage subgroups, the whole abdomen model maintained robust performance. It is known that there is a big difference between the early and advanced stages of EOC prognosis. Previous studies in cancer radiomic prognosis prediction tended to separate cancers into early and advanced stages for analysis (22, 23). However, it has not been realistic to determine the stage and pathological type of cancer before operations. Therefore, the ideal situation would be that regardless of the stage or pathological types of the patient, results regarding platinum sensitivity could be obtained by using only CE-MRI data. This is what our model delivers.

Additionally, the decision function value showed an association with the PFS of patients with EOC by Kaplan–Meier analysis (*P* <.001). ROC curves in PFS prediction indicated that the whole abdomen model had good prediction power in 1-year PFS yet was weakened in 2-year PFS predictions. Perhaps the decline in accuracy is due to the passing of time and more confounding factors (follow-up treatment, income, religious difference). Overall, the decision function value generated by the whole abdomen model was a good short-term prognostic indicator but weakened over time.

Deep learning is often referred to as a “black box” due to the lack of interpretability of the identified features (24). Huge potential benefits for clinical and fundamental research await those who can open the “black box” and make features more interpretable. In our study, features were made more accessible by creating visually appealing heat maps, generated over the last convolutional layer. Then, we analyzed the heat maps and incorporated medical knowledge into these invisible features. The high-response areas appeared to be the vasculature and peritoneal stroma in these heat maps, which suggested that the model was using features outside of the primary tumor as well as within. These regions were more relevant than the other areas as they attracted more attention to the whole abdomen model and consequently contained more platinum sensitivity features. In previous studies, peritumoral regions in cancer were valuable in estimating chemotherapy response (25, 26), and adding peritumoral regions led to increased AUC (27), which was consistent with our observations of the heat maps and might be explained by the fact that abundant vasculature and the micro-environment of the metastatic stroma might be related to platinum resistance and prognosis (28, 29).

At the same time, the severity of EOC metastasis in the peritoneal and mesenteric at CT seemed to be an indicator of significantly shorter PFS (30, 31). EOC cells typically spread within the peritoneal cavity with the dissemination of tumor cells into the peritoneal fluid, followed by implantation on the mesothelial linings of the omentum and other peritoneal surfaces that overlie connective and adipose tissues. A retrospective study that enrolled 46 women with HGSO found that the pattern of peritoneal involvement and the presence of mesenteric infiltration on pretreatment CT appear to be associated with prognostically relevant gene signatures (32). These indicated that due to the biological characteristics of intraperitoneal dissemination of EOC, features outside of the primary tumor were gene- and prognosis-related.

Therefore, it is unsatisfactory to only use the primary tumor as the VOI in prognosis prediction. Instead, features based on the whole abdomen were necessary for the deep-learning model to make a prediction. This may explain why the whole abdomen model achieved superior performance over the primary tumor model. However, this pattern may not be suitable for all cancers.

### Limitations

This research had various limitations. First, due to the limitation of sample size, this study lacked perspective and extensive data cohorts to ensure clinical applicability. Second, due to the retrospective nature, there was a lack of complete genetic data. Therefore, combining the genetic profiles and further explaining the deep learning features at the genetic level will become the focus of our future work.

## Conclusion

This study showed that deep learning can provide new MRI-based prognostic markers that have high sensitivity and specificity for platinum-sensitivity predictions of epithelial ovarian cancer (EOC). The volume of interest of the whole abdomen demonstrated stronger predictive power than the primary tumor lesion alone. These results represent a step forward for automated deep learning without any segmentation. This study conducted a meaningful exploration of deep-learning interpretability and pointed out the potential relationship between vascular density, tumor micro-environment, and platinum sensitivity of EOC as well as revealing the potential of deep learning visualization in promoting future clinical and basic research.

## Data availability statement

The raw data supporting the conclusions of this article will be made available by the authors, without undue reservation.

## Ethics statement

The studies involving human participants were reviewed and approved by the Institutional Review Boards of Sun Yat-sen Memorial Hospital. Written informed consent for participation was not required for this study in accordance with the national legislation and the institutional requirements.

## Author contributions

RL and HY had full access to all the data in the study and take responsibility for the integrity of the data and the accuracy of the data analysis. Concept and design: RL, HY, ZL, and YY. Acquisition, analysis, or interpretation of data: All authors. Drafting of the manuscript: RL, YY, QL, and HY. Critical revision of the manuscript for important intellectual content: All authors. Funding acquisition: HY. Administrative, technical, or material support: YY, ZL, and HY. Supervision: ZL and HY. All authors contributed to the article and approved the submitted version.

## Funding

This study was supported by grants 81972471 and 82073408 from the National Natural Science Foundation of China, grant 202206010078 and 202201020574 from the Guangzhou Science and Technology Project, grant 2018007 from the Sun Yat-Sen University Clinical Research 5010 Program, grant SYS-C-201801 from the Sun Yat-Sen Clinical Research Cultivating Program, grant A2020558 from the Guangdong Medical Science and Technology Program, grant 7670020025 from Tencent Charity Foundation, grant YXQH202209 from the Scientific Research Launch Project of Sun Yat-Sen Memorial Hospital.

## Conflict of interest

QY, JW, and LC are employed by Cells Vision Medical Technology Inc., Guangzhou, China.

The remaining authors declare that the research was conducted in the absence of any commercial or financial relationships that could be construed as a potential conflict of interest

## Publisher’s note

All claims expressed in this article are solely those of the authors and do not necessarily represent those of their affiliated organizations, or those of the publisher, the editors and the reviewers. Any product that may be evaluated in this article, or claim that may be made by its manufacturer, is not guaranteed or endorsed by the publisher.
