# Supplementary material for: Deep learning magnetic resonance imaging predicts platinum sensitivity in patients with epithelial ovarian cancer
Source: Front Oncol. 2022 Nov 23;12:895177. doi: 10.3389/fonc.2022.895177 (PMC9727155; doi:10.3389/fonc.2022.895177)
Supplement: Supplementary file 1 [file DataSheet_1.docx]

**Supplementary Online Content**

| **Methods S1** | Magnetic resonance imaging acquisition |
| --- | --- |
| **Methods S2** | Spatial and intensity normalization |
| **Methods S3** | Feature extraction |
| **Methods S4** | Feature decomposition |
| **Methods S5** | Classification |
| **Figure S1** | Performance of the primary tumor model for platinum-sensitive prediction |
| **Figure S2** | HIPEC and non-HIPEC subgroup analysis of the whole abdomen model |
| **Figure S3** | Stage subgroup analysis of the whole abdomen model |
| **Figure S4** | Pathological types subgroup analysis of the whole abdomen model |
| **Figure S5** | Surgery subgroup analysis of the whole abdomen model |
| **Table S1** | Magnetic resonance imaging scanning parameters for the patients |

**Table S2** Univariate analysis of clinic pathological characteristics with platinum sensitivity

**Table S3** Prediction performance of two models.

**Methods S1. Magnetic resonance imaging acquisition.**

In Sun Yat-sen Memorial Hospital of Sun Yat-sen University, patients underwent MRI scan using 1.5T or 3.0T scanners with 8-channel or 18-channel phased-array abdominal coils. At the time of scanning, axial fat-suppressed T2-weighted imaging (T2WI) sequence and axial diffusion-weighted imaging quantitatively measured apparent diffusion coefficients (DWI-ADC). Images were obtained with two b values (0 and 800 s/mm2) before contrast medium administration. An initial fat-saturated T1WI pre-contrast scan was collected before the scan of contrast-enhanced T1-weighted imaging (T1+C) images, and then T1+C images were acquired as 50~70 post contrast scans at intervals of 6~8 seconds following the intravenous injection of gadolinium contrast agent. A gadolinium-based agent (Magnevist; Bayer Healthcare, Berlin, Germany) was injected using an MR imaging compatible power injector at a rate of 3.5 ml/s and a dose of 0.2 ml/kg of body weight, followed by 20 ml saline flush with high-pressure injector.

**Methods S2. Spatial and intensity normalization**

Spatial and intensity normalization were applied to the input VOI. First, every sequence in our dataset was re-sampled into an identical spacing of height=0.8, width=0.8, depth=3.0. These values were selected from the approximate median value in our dataset, and linear interpolation was used in the re-sampling. Second, the intensity within each single volume was normalized to have zero mean and unit variance, intensity value **ν_i_** was normalized to **ν_i_'** by using the mean **ν_m_** and standard deviation **ν_std_**of the non-zero voxels in the individual sequence as:

$$\nu_{i}'=\frac{\nu_{i}-\nu_{m}}{\nu_{\mathrm{std}}}$$

The **ν_i_** represents the value of the i-th non-zero voxel before normalization and **νi**' is the value calculated.

**Methods S3. Feature extraction**

A pre-trained 3-dimensional convolution neural network 3D-ResNet Med3D (version 4.10.2) https://www.slicer.org/) was adopted to extract features from the pre-processed chosen VOI. Transfer learning is a machine learning technique that allows a model trained on one task to be transferred to a second task.[1]^-^[2] Normally, when applying transfer learning to a small-scale dataset, certain layers are preferably frozen to reduce the destruction of information contained in the weights which were well trained on a much larger and more general dataset.

We took the pre-trained Med3D network, which was originally trained on segmentation tasks on various medical image datasets and extracted useful, general three-dimensional features from the medical image source[1]. We then removed the up-sample layers and a global average pooling layer was added right after the last convolution layer so that 512 features could be extracted from each MRI sequence regardless the size of the input sequence. Since there were two MRI sequences for each patient, the two 512-features were concatenated to acquire a 1,024-dimension feature.

**Methods S4. Feature decomposition**

Principal component analysis (PCA) [3] was performed to reduce model complexity and noise, to extract the most informative characteristics from the features, and to avoid overfitting caused by high dimensional sparse features and the small number of training samples. This technique is one of the most commonly used dimensionality-reduction methods. It compressed the dimensionality of the features by factorizing a vector of features into three matrices: two unitary matrices (U, V) and one diagonal matrix (S) of singular values such that A = USV [3], and performed a linear transformation to map the original features to a lower-dimensional space and maximize the variance of the data in the lower-dimension.

Whitening [4] was used in PCA, so that the features could be less correlated with each other and maintain the same variance. Data were labelled into two groups according to platinum-sensitive or resistant, and we then fit the PCA with patients accordingly. The number of components was carefully chosen so that the amount of explained variance was higher than 80%.

**Methods S5. Classification**

After the dimensionality reduction, a Support Vector Machine (SVM) was fit on the data produced by the PCA and trained with RBF kernel to predict the target label (platinum-sensitive or resistant). Although in practice, SVMs tends to be resistant to overfitting, the optimal combination of the penalty term C and the kernel coefficient value γ still need to be selected. Smaller γ means larger RBF bandwidths and a smoother decision boundary and vice versa. And a smaller C has a higher tolerance to misclassified data and prefers larger margins. Grid search technique was used to decide the hyper-parameters. A few potential hyper-parameter combinations were looped through and cross-validated on the training set to see how well the model was generalized. As per the result, C=50, γ=0.5 were selected to be optimal.

Then, the SVM on the entire training dataset was retrained. Finally, the decision function value of the SVM predicted how likely the patient was to be platinum-sensitive.


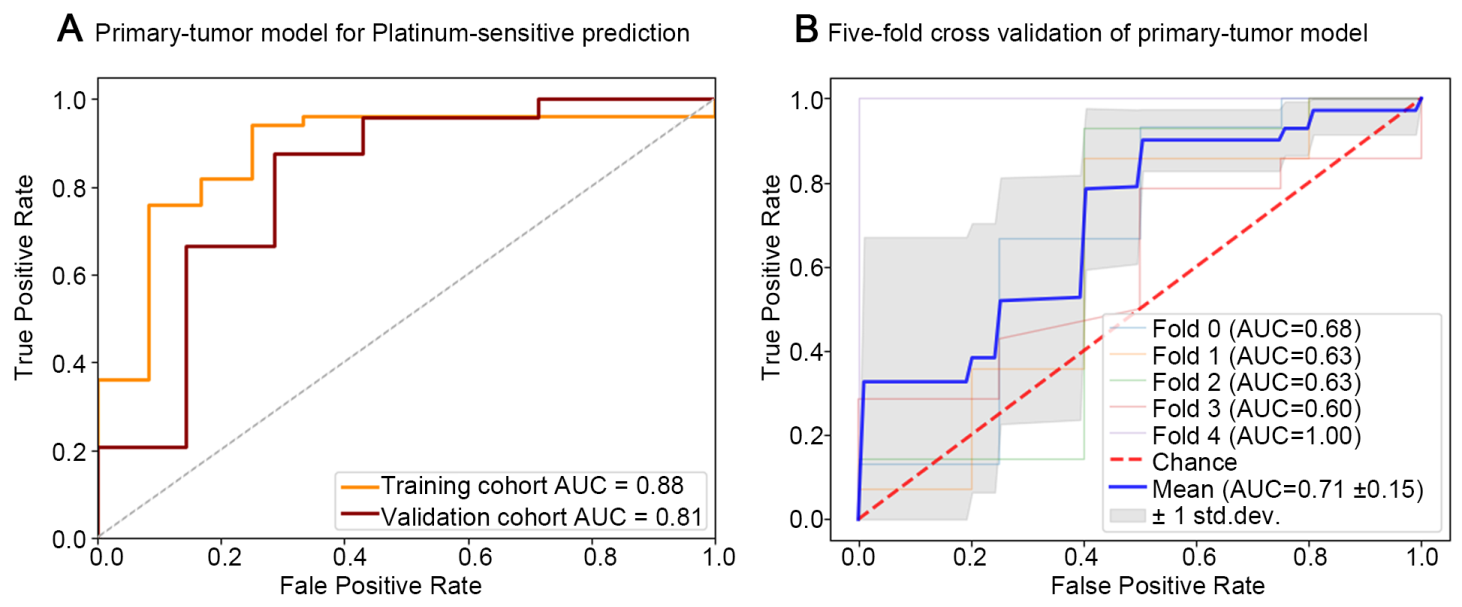

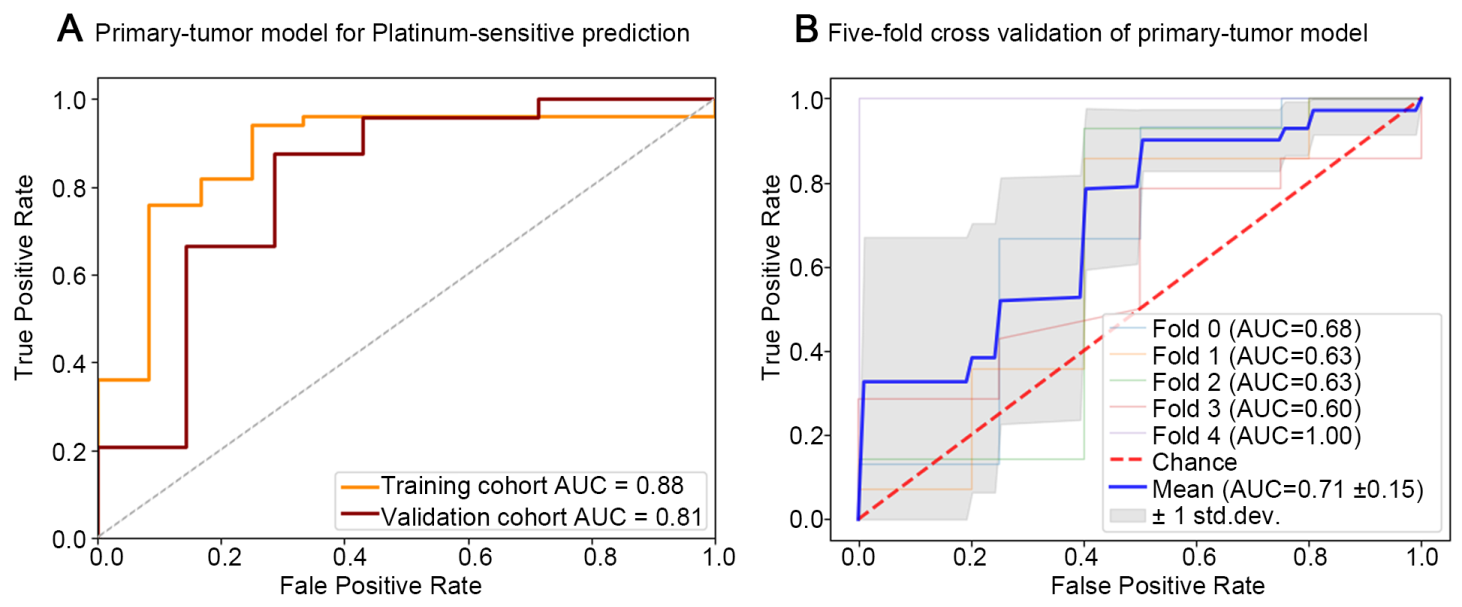


**Figure S1**. Performance of the primary tumor model for platinum-sensitive prediction. Performance of the primary tumor model for predicting platinum-sensitivity, in training and validation cohorts(A), and 5-fold cross-validation (B). AUC, area under the receiver operating characteristics curve.


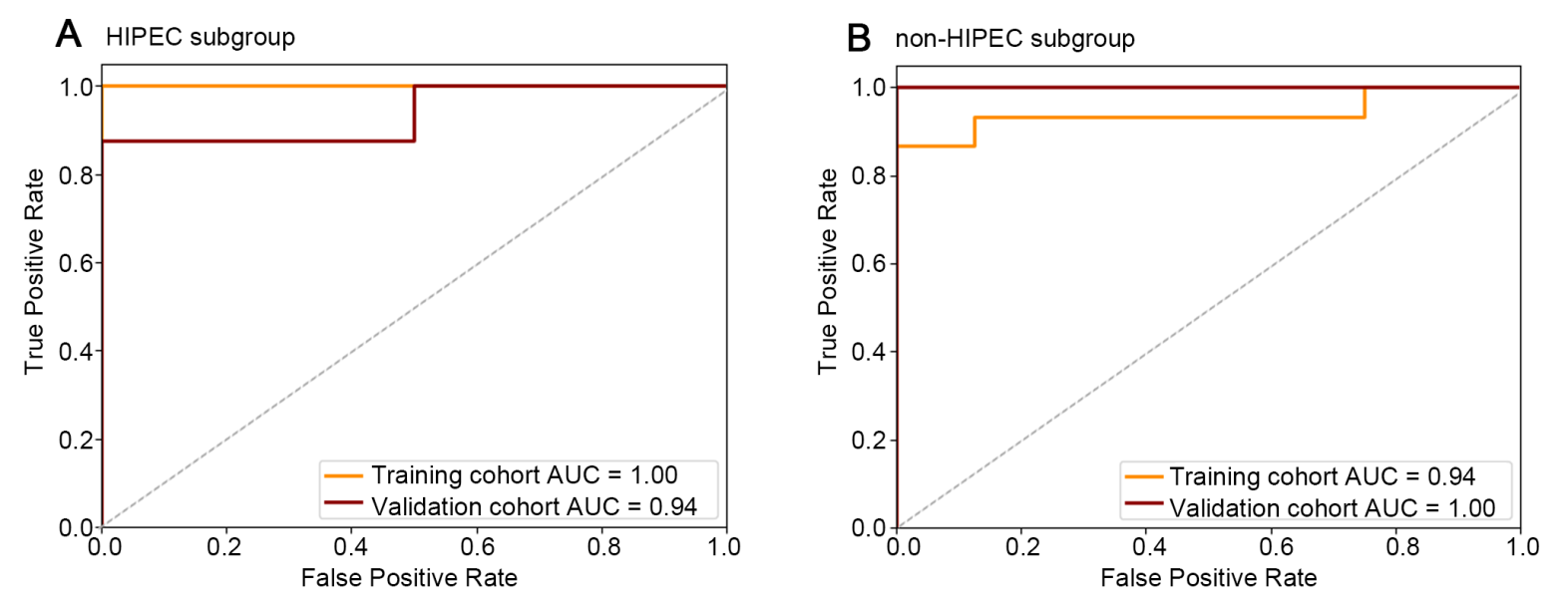

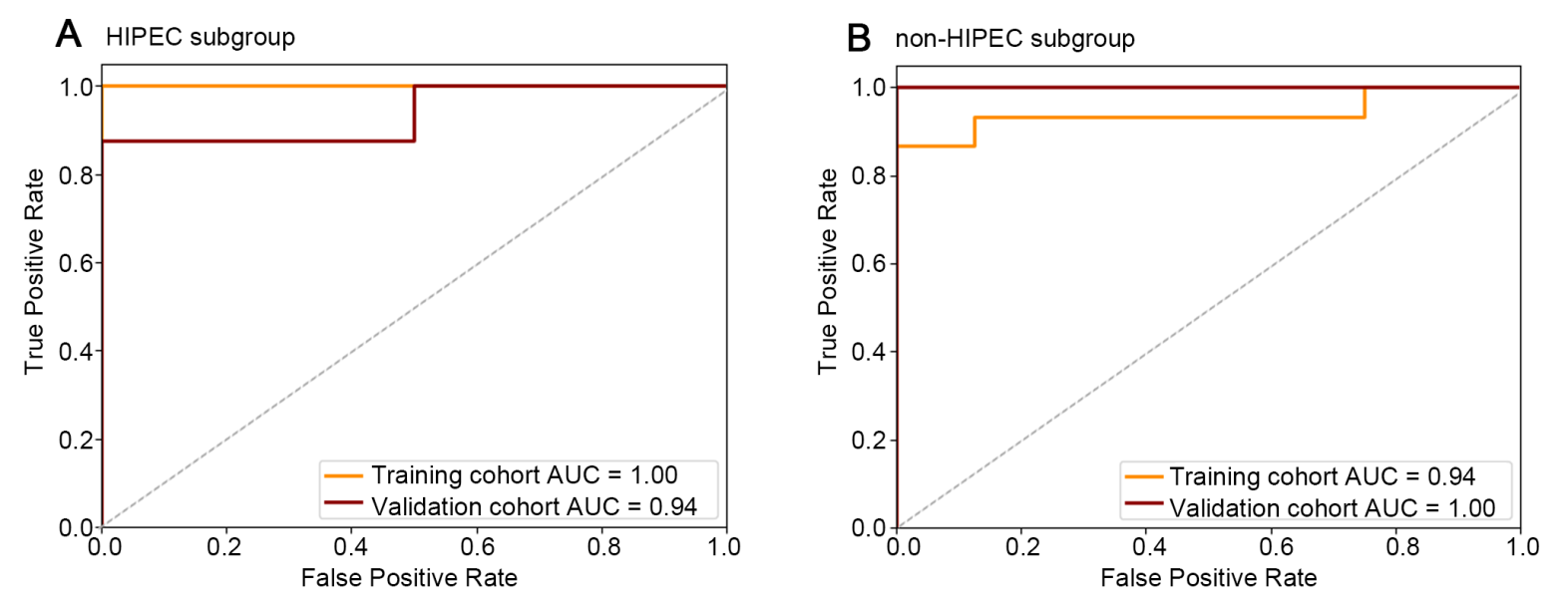


**Figure S2.** HIPEC and non-HIPEC subgroup analysis of the whole abdomen model

Performance of the whole abdomen model for predicting platinum-sensitivity in (A) HIPEC and (B) non-HIPEC subgroup analysis. AUC, area under the receiver operating characteristics curve. HIPEC, Hyperthermic intraperitoneal chemotherapy.


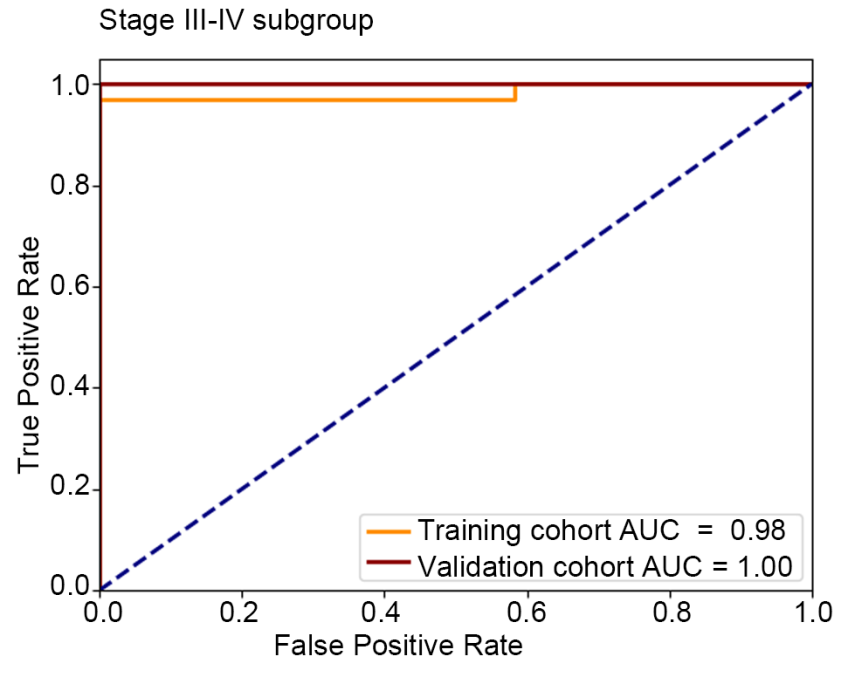


**Figure S3.** Stage subgroup analysis of the whole abdomen model. Performance of the whole abdomen model for predicting platinum-sensitivity in stage III-IV subgroup analysis. AUC, area under the receiver operating characteristics curve.


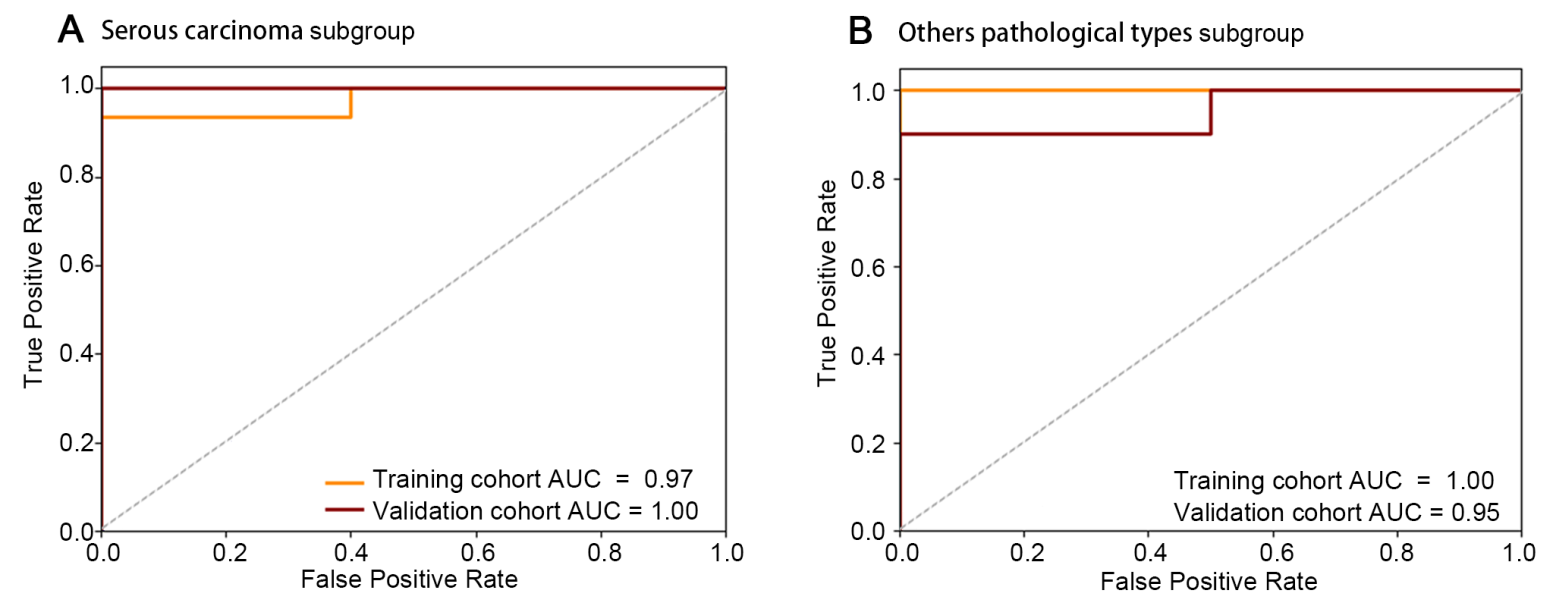

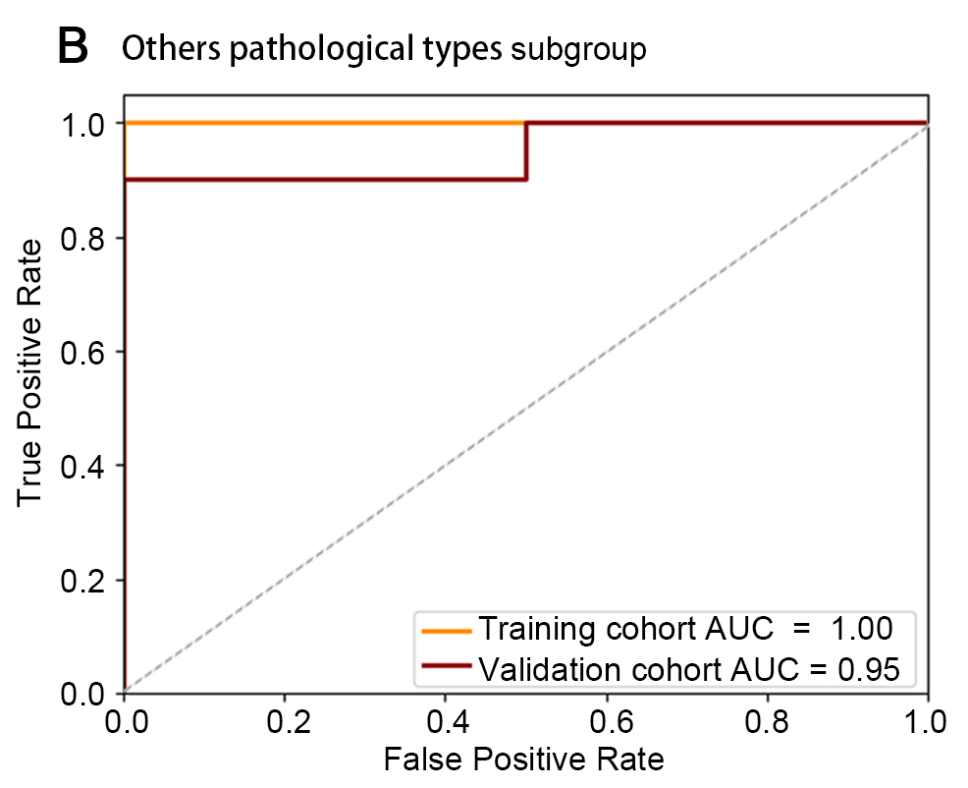


**Figure S4.** Pathological type subgroup analysis of the whole abdomen model

Performance of the whole abdomen model for predicting platinum-sensitivity in (A) serous adenocarcinoma and (B) other pathological types subgroup. AUC, area under the receiver operating characteristics curve.

.


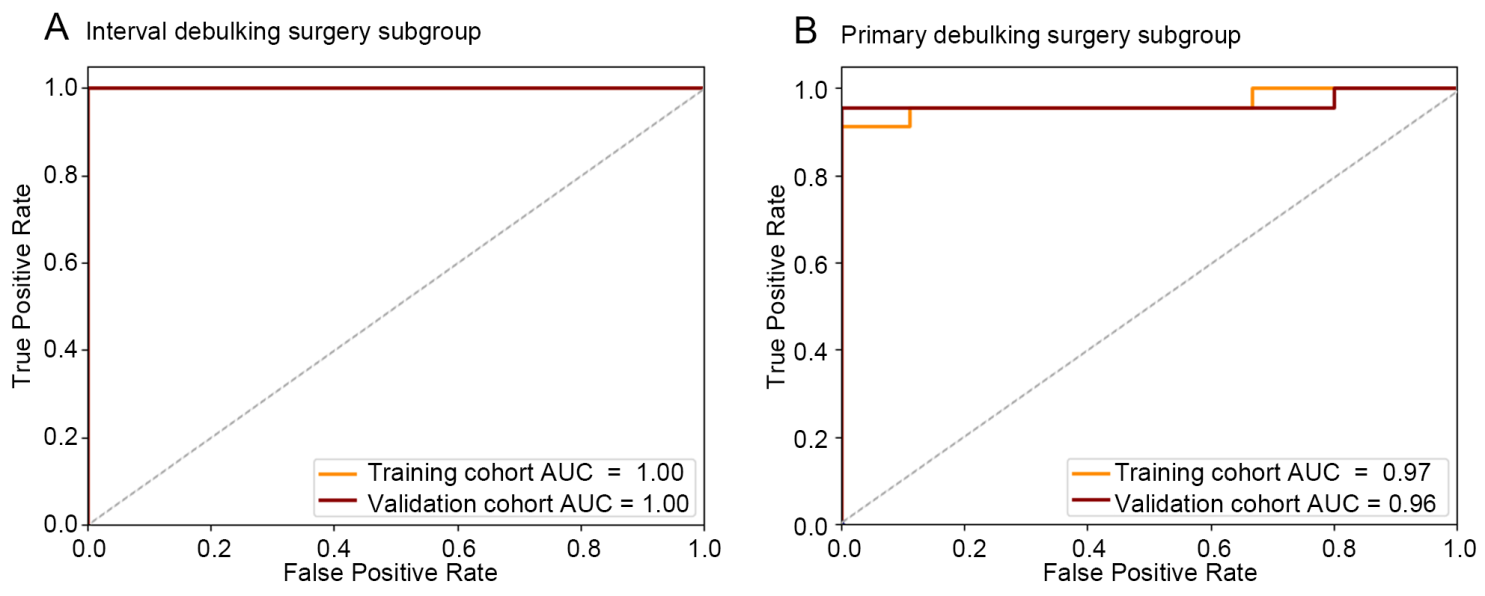

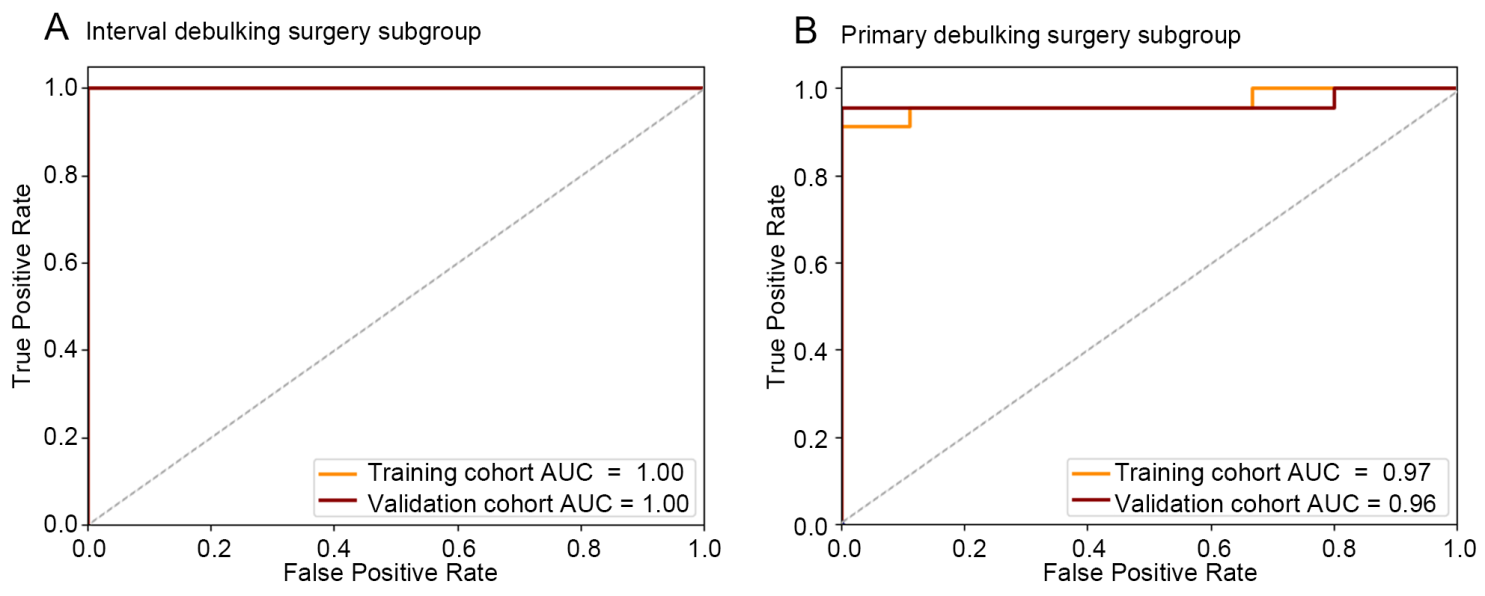


**Figure S5.** Surgery subgroup analysis of the whole abdomen model

Performance of the whole abdomen model for predicting platinum-sensitivity in (A) interval debulking surgery and (B) primary debulking surgery subgroup in training and validation cohorts. AUC, area under the receiver operating characteristics curve.

**Table S1.** Magnetic resonance imaging scanning parameters for the patients

| Hospital | Scanner | Sequence | TR/TE  (ms) | FOV  (mm) | Matrix | Slice Thickness (mm) | Slice Gap  (mm) | Flip Angle |
| --- | --- | --- | --- | --- | --- | --- | --- | --- |
| SYSMH | Philips 1.5T  (Achieva) | T2WI | 3600/96 | 300×260 | 320×288 | 4 | 0.8 | 90° |
|  |  | T1+C | 3.3/1.54 | 300×260 | 320×288 | 1 | 0 | 10° |
|  | Philips 3.0T  (Ingenia) | T2WI | 3100/110 | 300×260 | 320×288 | 4 | 0.4 | 90° |
|  |  | T1+C | 5.9/2.1 | 300×260 | 320×288 | 3 | 0 | 10° |
|  | Siemens 1.5T  (Avanto) | T2WI | 2800/100 | 300×260 | 320×288 | 4 | 0.8 | 160° |
|  |  | T1+C | 7.03/2.39 | 300×260 | 320×288 | 1 | 0 | 10° |
|  | Siemens 3.0T  (Skyra) | T2WI | 3200/119 | 300×260 | 320×288 | 4 | 0.4 | 120° |
|  |  | T1+C | 3.97/1.26 | 300×260 | 320×288 | 1 | 0 | 10° |

Abbreviations: FOV, field of view; TR, repetition time; TE, echo time; T1+C, contrast-enhanced T1-weighted imaging; T2WI, T2-weighted imaging

**Table S2.** Univariate analysis of clinicopathological characteristics with platinum sensitivity.

|  | Training cohort | | | |
| --- | --- | --- | --- | --- |
| Characteristics | Platinum-sensitive No. (%) | Platinum-resistant No. (%) | OR(95%CI) | *P* value |
| Age, years |  |  |  |  |
| ＜40 | 10 (20) | 1 (8) | ref |  |
| ≥40 | 40 (80) | 11 (92) | 0.36 (0.04, 3.16) | .36 |
| HIPEC |  |  |  |  |
| Non | 30 (60) | 8 (67) | ref |  |
| ≥1 cycle | 20 (40) | 4 (33) | 1.33 (0.35, 5.03) | .67 |
| FIGO stage^a^ |  |  |  |  |
| I-II | 17 (45.7) | 0 (0) |  |  |
| III-IV | 19 (54.3) | 26 (100) | - | .00 |
| Histologic classification |  |  |  |  |
| Serous carcinoma | 32 (64) | 7 (58) | ref |  |
| Others | 18 (36) | 5 (42) | 0.79 (0.22, 2.85) | .72 |
| Type of surgery |  |  |  |  |
| IDS | 5 (10) | 3 (25) | ref |  |
| PDS | 45 (90) | 9 (75) | 3.00 (0.61, 14.86) | .18 |

Abbreviations: OR, odds ratio; CI, confidence interval; HIPEC, hyperthermic intraperitoneal chemotherapy; PDS, primary debulking surgery; IDS, interval debulking surgery.

^a^ 2018 FIGO staging.

**Table S3.** Prediction performance of two models.

| **Deep Learning Model** | | AUC (95%CI) | Accuracy | Sensitivity | Specificity |
| --- | --- | --- | --- | --- | --- |
| **Primary tumor model** | Training Cohort | 0.88 (0.79, 0.97) | 0.87 | 90% | 75% |
|  | Validation Cohort | 0.81 (0.65, 0.97) | 0.81 | 88% | 57% |
|  | k^a^-fold cross validation | 0.71 (0.56, 0.86) | 0.73 | 77% | 64% |
| **Whole abdomen model** | Training Cohort | 0.97 (0.93, 1.00) | 0.95 | 96% | 92% |
|  | Validation Cohort | 0.98 (0.93, 1.00) | 0.97 | 96% | 100% |
|  | k-fold cross validation | 0.97 (0.92, 1.00) | 0.95 | 96% | 89% |

Abbreviations: AUC, area under the receiver operating characteristics curve; CI, confidence interval.

^a^ K=5

**References:**

1 Chen S, Ma K, Zheng Y. (2019). Med3D: Transfer Learning for 3D Medical Image Analysis (1904.00625, pp.): arXiv:1904.00625;.[online] Available: https://arxiv.org/abs/1904.00625. (Reprinted. doi: https://arxiv.org/abs/1904.00625.

2 Tan C, Sun F, Kong T, Zhang W, Yang C, Liu C. (2018-01-01). *A survey on deep transfer learning*, 2018. Springer, p 270-279

3 Jolliffe IT, Cadima J (2016) Principal component analysis: a review and recent developments. Philosophical Transactions of the Royal Society A: Mathematical, Physical and Engineering Sciences 374:20150202

4 Jégou HAOC. (2012-01-01). *Negative evidences and co-occurences in image retrieval: The benefit of PCA and whitening*, 2012. Springer, p 774-787
